# Tumeurs occipitales: une urgence médicale ou chirurgicale?

**DOI:** 10.11604/pamj.2014.17.121.3699

**Published:** 2014-02-19

**Authors:** Rhizlane Berrady, Wafaa Bono

**Affiliations:** 1 Service De Médecine Interne, CHU Hassan II Fès, Faculté de Médecine de Fès, Route sidi Hrazem,30000 Fès, Maroc

**Keywords:** Tumeur occipitale, urgence médicale, plasmocytome, occipital tumor, medical emergency, plasmacytoma

## Image en médicine

Le plasmocytome est une tumeur constituée d'une prolifération monoclonale de plasmocytes malins, pouvant rentrer ou non dans le cadre d'un myélome. Nous rapportons le cas d'un patient âgé de la cinquantaine, sans antécédents médicaux particuliers, ayant consulté pour tuméfaction de siège occipitale augmentant progressivement de volume. L'examen clinique ne note aucun signe de focalisation neurologique. Un bilan radiologique fait ayant objectivé une lyse osseuse intéressant la table interne et externe de l'os occipitale. Un bilan biologique initial a montré une pancytopénie. L’étude cytologique du myélogramme a mis en évidence une plasmocytose médullaire. Le diagnostique du myélome multiple avec plasmocytome occipital est confirmé par l’électrophorèse des protides qui a objectivé un pic monoclonale au niveau des gammaglobulines. Le patient a reçu une chimiothérapie. L’évolution est marquée par l'affaissement total de la masse tumorale.

**Figure 1:**
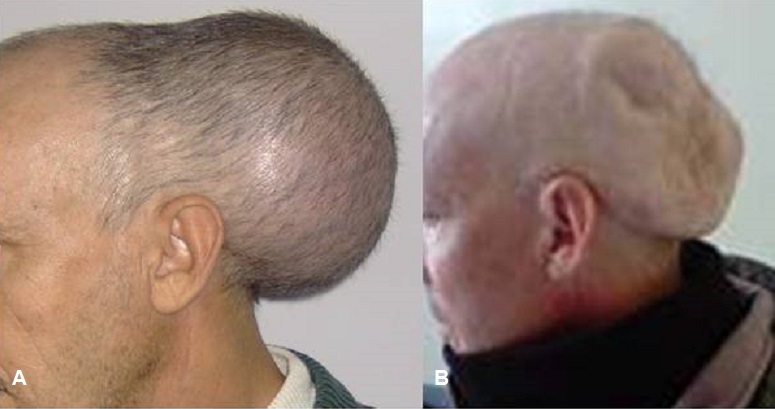
Tuméfaction occipitale (A) avant et (B) après traitement medical

